# Clinical characteristics of synchronous and metachronous superficial esophageal squamous cell carcinoma during surveillance after endoscopic submucosal dissection

**DOI:** 10.1007/s00464-026-12816-3

**Published:** 2026-04-20

**Authors:** Marina Kuroda, Yohei Ikenoyama, Hiroto Suzuki, Aiji Hattori, Misaki Nakamura, Yasuhiko Hamada, Noriyuki Horiki, Hayato Nakagawa

**Affiliations:** 1 Department of Gastroenterology and Hepatology, Mie University Graduate School of Medicine, Mie University Hospital, 2-174 Edobashi, Tsu, Mie 514-8507 Japan; 2 Department of Endoscopy, Mie University Graduate School of Medicine, Tsu, Japan

**Keywords:** Esophageal squamous cell carcinoma, Endoscopic submucosal dissection, Metachronous neoplasms, Clinicopathologic characteristics

## Abstract

**Background:**

Although endoscopic submucosal dissection (ESD) is the standard treatment for superficial esophageal squamous cell carcinoma (SESCC), the clinical characteristics of synchronous and metachronous (secondary) lesions detected during follow-up remain unclear. This study aimed to compare the clinicopathologic characteristics and outcomes of initial and secondary SESCC lesions.

**Methods:**

This single-center study analyzed 307 esophageal squamous cell carcinoma lesions in 188 patients who underwent ESD as their initial treatment between January 2005 and January 2025. The lesions were categorized as initial (*n* = 208) or secondary (*n* = 99) based on their timing of detection. Clinicopathological features and short-term outcomes were compared between the two groups.

**Results:**

Patients with multiple lesions were younger and had a higher prevalence of prior head and neck cancer and a higher prevalence of Lugol-voiding lesion grades B/C. The median time to detection of secondary lesions was 18 months. Secondary lesions were smaller (< 20 mm: 88% vs. 44%) and shallower (epithelial/lamina propria mucosa: 89% vs. 74%) than initial lesions. Multivariate analysis revealed that tumor size < 20 mm (odds ratio 0.12, 95% confidence interval 0.06–0.23) and epithelial/lamina propria mucosa invasion depth (odds ratio 0.37, 95% confidence interval 0.17–0.84) were independently associated with secondary lesions. In secondary lesions, curative resection rates were higher (84% vs. 68%, *p* < 0.005) and complications less frequent (12% vs. 34%, *p* < 0.001). Esophageal stenosis occurred less frequently in secondary lesions. The cumulative incidences of secondary lesions were 10.2%, 18.9%, and 28.2% at 1, 3, and 5 years, respectively.

**Conclusion:**

Secondary SESCC lesions tend to be smaller and shallower, enabling higher curative resection rates and lower complication risks. These findings suggest that secondary lesions are more amenable to curative ESD when detected at an early stage. Regular surveillance after ESD facilitates early detection of secondary lesions and their curative resection.

**Graphical Abstract:**

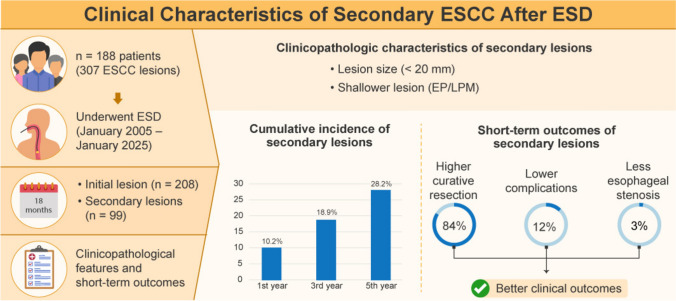

**Supplementary Information:**

The online version contains supplementary material available at 10.1007/s00464-026-12816-3.

Esophageal cancer ranks as the eighth most prevalent cancer worldwide and the sixth leading cause of cancer-related mortality [1]. Esophageal squamous cell carcinoma (ESCC) remains the predominant histologic type, particularly in Southern and Eastern Africa and Central Asia, where it accounts for most cases [2, 3]. In Japan, 80–90% of esophageal cancers are ESCC, and both incidence and mortality have increased with the aging population [4]. Early detection is crucial for achieving favorable treatment outcomes, as ESCC can progress rapidly and requires surgical intervention once it invades the submucosal layer. Therefore, early diagnosis leading to indications for endoscopic submucosal dissection (ESD) and treatment with ESD is important [5, 6].

The optimal post-ESD management strategy has not been fully established and remains a subject of ongoing debate. A unique feature of ESCC is its propensity to develop multiple lesions over time [7], necessitating repeated interventions and long-term surveillance. Similarly, high rates of metachronous recurrence after endoscopic treatment have been reported in Barrett’s esophageal adenocarcinoma [8, 9], for which eradication of the background Barrett’s mucosa using radiofrequency ablation in addition to local resection is recommended [10, 11]. However, eradication therapy is not generally recommended for ESCC, and routine endoscopic surveillance remains the standard management approach. To establish appropriate management after initial ESD, clarifying the clinicopathologic features of synchronous and metachronous cancers is crucial. However, the natural history and clinical behavior of these lesions detected during follow-up remain poorly understood. To address this issue, this study compared the clinicopathologic characteristics and treatment outcomes of initial lesions with those of synchronous and metachronous lesions to clarify their distinct clinical features.

## Materials and methods

### Study design and patients

**Fig. 1:**
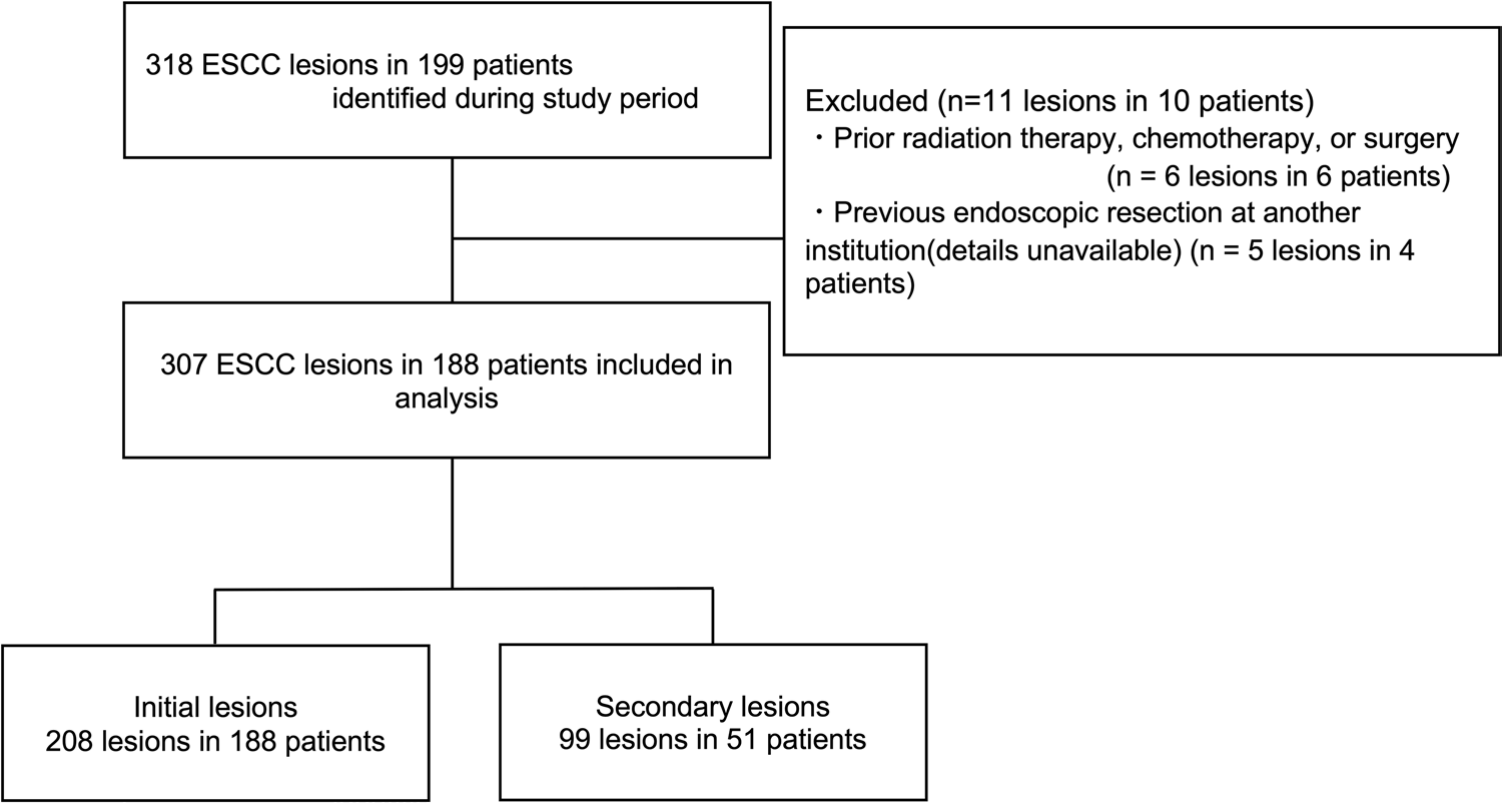
Flowchart of patient and lesion selection. Of the 318 lesions identified, 307 lesions were analyzed after exclusions, including 208 initial and 99 secondary lesions. *ESCC*, esophageal squamous cell carcinoma; *ESD*, endoscopic submucosal dissection

This single-center retrospective study included patients with ESCC diagnosed endoscopically between January 2005 and January 2025. During the study period, 318 ESCC lesions were identified in 199 patients who underwent ESD as the initial treatment for SESCC at our institution. Patients were excluded if they had received radiation therapy, chemotherapy, or surgical treatment for ESCC prior to ESD (six lesions in six patients) or if they had undergone previous endoscopic resection at another institution (five lesions in four patients). After excluding 11 lesions in 10 patients, a total of 307 lesions in 188 patients were included in the final analysis. Lesions were subsequently classified into initial and secondary (synchronous and metachronous) categories, as illustrated in the study flowchart (Fig. 1).

### Endoscopic resection

Endoscopic resection of SESCC was performed using ESD. Narrowband imaging and iodine staining were employed to determine lesion extent, with markings placed a few millimeters beyond the lesion margins. Local injection of glycerol or hyaluronic acid into the submucosa was subsequently performed, followed by mucosal incision and submucosal dissection [12]. The following knives were frequently used: dual knife (KD-650; Olympus, Tokyo, Japan), MP knife (KD-655; Olympus, Tokyo, Japan), SB knife Jr (MD-47703W; Sumitomo Bakelite, Tokyo, Japan), and IT-Knife nano (KD-612; Olympus).

ESD was indicated for non-circumferential lesions of clinical T1a-epithelial (EP)/lamina propria mucosa (LPM) cancer without lymph node or distant metastasis (N0M0), as well as for circumferential lesions with a longitudinal resection length of ≤ 50 mm [13–15]. Clinical T1a-muscularis mucosae cancer is considered a relative indication for ESD [13].

After ESD, curative status was determined based on histological findings of the resected specimen. In ESCC, pathological T1a-EP/LPM (pT1a-EP/LPM) lesions negative for vascular invasion and with negative margins were considered curative resections, as lymph node metastasis is infrequent [14, 15]. Patients meeting these criteria were defined as curative cases.

Secondary lesions were treated with ESD, argon plasma coagulation (APC), or surgical resection. APC was performed for lesions that were clinically diagnosed as clinical EP/LPM (cEP/LPM) and considered to have a high risk for ESD. Pathological assessment was not performed for the lesions treated with APC. Lesions suspected to have submucosal invasion were treated with surgical resection.

### Post-ESD surveillance endoscopy

Follow-up endoscopic examinations were performed using a high-resolution, high-intensity lighting system (EVIS LUCERA ELITE [2012] and EVIS X1 [2020]; Olympus, Tokyo, Japan) and endoscopes (GIF-H260, GIF-H290Z [2015], and GIF-XZ1200 [2021]; Olympus Medical Systems, Tokyo, Japan). The interval between follow-up examinations was approximately 6 months. In this retrospective cohort, the surveillance intervals were not strictly protocolized based on risk categories. The follow-up interval was defined as the period between two endoscopies, whereas the follow-up period referred to the time from the initial ESD to the end of follow-up at our hospital.

Endoscopic assessment was basically performed using white-light imaging and narrowband imaging. In many cases, Lugol’s test was used to evaluate recurrence risk, unless the patient was allergic to iodine. Multiple Lugol-voiding lesions (LVLs) were defined as follows: A, no LVL; C, ≥ 10 LVLs per field of view; and B, between A and C [16].

### Definitions

Patients were categorized according to the number of ESCC lesions. Those with only one lesion were assigned to the single-lesion group, whereas individuals with more than one lesion, including synchronous lesions, were included in the multiple-lesion group.

Lesions were further categorized as initial or secondary. Initial lesions were defined as lesions detected at the time of the first diagnosis, including concurrent lesions. Secondary lesions were defined as synchronous and metachronous lesions, which were identified as additional SESCCs detected at anatomically distinct locations from the index lesion during the clinical course. Local recurrence—defined as a lesion arising within the post-ESD scar in cases with positive or indeterminate horizontal margins—was not observed in this study.

Complications were defined as follows: perforation was diagnosed when endoscopically confirmed during or after ESD; pneumomediastinum was defined as the presence of emphysema on chest radiographs or computed tomography; bleeding was defined as uncontrolled hemorrhage requiring termination of ESD or endoscopic hemostasis during hospitalization [17]; fever or pneumonia was defined as a body temperature ≥ 38 °C or a diagnosis of pneumonia; and stenosis was defined as subjective obstructive symptoms with difficulty passing an oral scope with a diameter of 9.8–9.9 mm [18].

### Statistical analysis

This single-center retrospective observational cohort study was reported in accordance with the STROCSS 2021 guidelines. Statistical analyses were performed by the authors with experience in medical statistics. Fisher’s exact test or the chi-squared test was used to examine categorical variables. Continuous variables were compared between groups using parametric or non-parametric tests, depending on data distribution, and presented as median values with interquartile ranges (IQRs).

To identify clinicopathologic characteristics independently associated with secondary lesions, logistic regression analysis was performed. The dependent variable was lesion category (initial vs. secondary). Variables with a *p*-value < 0.10 in univariate analyses were entered into the multivariate logistic regression model. Odds ratios with 95% confidence intervals (CIs) were calculated. The cumulative incidence of secondary lesions was calculated using the Kaplan–Meier method. Analyses were performed using EZR version 1.27 (Saitama Medical Center, Jichi Medical University, Japan) [19] and R (version 4.2.0; R Foundation for Statistical Computing, Vienna, Austria). Two-sided *p*-value < 0.05 was considered significant.

### Ethics approval

This study was conducted in accordance with the 1964 Declaration of Helsinki and its later amendments. The protocol was reviewed and approved by the institutional review board of our hospital (IRB No. H2021-116).

## Results

### Baseline characteristics of enrolled patients with ESCC (*n* = 188)

Table 1 summarizes the baseline characteristics of patients with ESCC (*n* = 188). The study population was categorized into single- (*n* = 123) and multiple-lesion (*n* = 65) groups. Differences in patient backgrounds were observed between the groups. Patients in the multiple-lesion group were younger at the time of first onset than those in the single-lesion group (median age, 67 [63–72] vs. 70 [66–76] years, *p* = 0.010). Additionally, they had a higher prevalence of prior head and neck cancer (37% vs. 9%, *p* < 0.001) and a higher proportion of LVL grade B/C (91% vs. 71%, *p* < 0.001).

**Table 1:** Baseline characteristics of the study patients (*n* = 188)

	Single-lesion group (*n* = 123)	Multiple-lesion group (*n* = 65)	*p-*value
Age, years, median (IQR)	70 (66–76)	67 (63–72)	**0.010**
Sex, *n* (%)			0.809
Male	110 (89)	57 (88)	
Female	13 (11)	8 (12)	
Smoking, brinkman index ≥ 600, *n* (%)	66 (54)	40 (62)	0.354
Alcohol consumption ≥ 300 g of ethanol/week, *n* (%)	34 (28)	24 (37)	0.245
Previous head and neck cancer, n (%)	11 (9)	24 (37)	** < 0.001**
History of gastrectomy, n (%)	13 (11)	9 (14)	0.634
LVL grade, n (%)^a^			** < 0.001**
A	28 (23)	3 (5)	
B	68 (55)	27 (42)	
C	20 (16)	32 (49)	
Follow-up duration, months, median (IQR)	55 (16–86)	68 (47–114)	**0.0068**

*ESCC* esophageal squamous cell carcinoma, *IQR* interquartile range, *LVL* Lugol-voiding lesions

^a^LVL grades were graded into three categories (A = no lesion; B = 1–9 lesions; C ≥ 10 lesions)

Bold is to emphasize *p* < 0.05

The median time to detection of secondary lesions in the multiple-lesion group was 18 months (IQR, 4–38). The median surveillance interval during that period was 5.25 months (IQR, 1.95–8.23), defined as the average interval between follow-up endoscopies until the next lesion was identified. The median follow-up duration was 55 months (IQR, 16–86) in the single-lesion group and 68 months (IQR, 47–114) in the multiple-lesion group (p = 0.0068).

All initial lesions, comprising 208 lesions in 188 patients, were treated with ESD. Overall, 99 secondary lesions were identified in 51 patients, of which 90 lesions in 48 patients were treated with ESD, seven lesions in six patients were treated with APC, and two lesions required surgical resection.

### Comparison of the characteristics of initial and secondary lesions

Secondary cancers exhibited a significantly lower proportion of lesions ≥ 20 mm (12% vs. 56%, *p* < 0.001) and of lesions involving more than half of the esophageal circumference (23% vs. 51%, *p* < 0.001) compared with initial lesions. Secondary lesions were more frequently confined to pEP/LPM (89% vs. 74%, *p* = 0.003) (Table 2).

**Table 2:** Comparison of the characteristics of initial and secondary lesions

	Initial lesions (*n* = 208)	Secondary lesions (*n* = 99)	*p*-value
Macroscopic classification, *n* (%)			0.625
Protruding type	16 (8)	7 (7)	
Superficial type	65 (31)	36 (36)	
Excavated type	127 (61)	54 (55)	
Advanced type	0 (0)	2 (2)	
Location of lesion, *n *(%)			0.641
Ce, Ut	19 (9)	12 (12)	
Mt	122 (59)	54 (55)	
Lt, Ae	67 (32)	33 (33)	
Diameter of ≥ 20 mm, *n* (%)	116 (56)	12 (12)	** < 0.001**
Circumference involvement ≥ 1/2, *n* (%)	105 (51)	23 (23)	** < 0.001**
Depth of invasion, *n* (%)			**0.003**
EP/LPM	154 (74)	82 (89)	
MM/SM	53 (26)	10 (11)	

*Ce* cervical esophagus, *Ut* upper thoracic esophagus, *Mt* MIDDLE thoracic esophagus, *Lt* lower thoracic esophagus, *Ae* abdominal esophagus, *EP* Epithelium, *LPM* Lamina Propria Mucosae, *MM* Muscularis Mucosae, *SM* Submucosa

Bold is to emphasize *p* < 0.05

### Multivariate analysis comparing clinicopathologic characteristics between initial and secondary lesions.

Multiple logistic regression analysis identified tumor size < 20 mm (odds ratio [OR] = 0.12, 95% CI: 0.06–0.23, *p* < 0.001) and EP/LPM depth of invasion (OR = 0.37, 95% CI: 0.17–0.84, *p* = 0.017) as independent factors associated with secondary lesions. Circumferential involvement was not significantly associated with secondary lesions (*p* = 0.394) (Table 3).

**Table 3:** Multivariate analysis comparing clinicopathologic characteristics between initial and secondary lesions

	Univariate OR (95% CI)	*p*-value	Multivariate OR (95% CI)	*p*-value
Diameter of ≥ 20 mm, *n* (%)	**0.11 (0.06–0.21)**	** < 0.001**	**0.12 (0.06–0.23)**	** < 0.001**
Circumference involvement ≥ 1/2, *n* (%)	**0.30 (0.17–0.51)**	** < 0.001**	0.76 (0.40–1.43)	0.394
Depth of invasion (MM/SM), *n* (%)	**0.35 (0.17–0.73)**	**0.005**	**0.37 (0.17–0.84)**	**0.017**

*OR* odds ratio, *CI* confidence interval, *MM* muscularis mucosae, *SM* submucosa

Bold is to emphasize *p* < 0.05

### Comparison of short-term results between initial and secondary lesions

The curative resection rate was significantly higher in secondary lesions than in initial lesions (84% vs. 68%, *p* < 0.005). Overall complications were significantly less frequent in secondary lesions (12% vs. 34%, *p* < 0.001), with a notably lower rate of esophageal stenosis (3% vs. 16%, *p* = 0.001) (Table 4).

**Table 4:** Comparison of short-term results between initial and secondary lesions

	Initial lesions (*n* = 208)	Secondary lesions (*n* = 99)	*p*-value
HM, *n* (%)	18 (9)	5 (6)	0.480
VM, *n* (%)	1 (1)	1 (1)	0.515
LyV, *n* (%)	8 (4)	4 (4)	1.00
Curative resection, *n* (%)	140 (68)	76 (84)	** < 0.005**
Additional treatment, *n* (%)	20 (10)	3 (3)	0.077
Surgery	13 (6)	2 (2)	
Chemoradiotherapy	3 (1)	0 (0)	
Chemotherapy	1 (1)	0 (0)	
Radiotherapy	3 (1)	1 (1)	
Complications, *n* (%)	71 (34)	12 (12)	** < 0.001**
Bleeding	11 (5)	4 (4)	0.781
Perforation	3 (2)	0 (0)	0.554
Esophageal stenosis	33 (16)	3 (3)	**0.001**
Infection/pneumonia	19 (9)	3 (3)	0.060
Pneumomediastinum	2 (1)	0 (0)	1.00

*HM* horizontal margin, *VM* vertical margin, *LyV* lymphatic invasion/vascular invasion

Bold is to emphasize *p* < 0.05

Details regarding the management and clinical outcomes of treatment-related complications are summarized in Supplementary Table 1.

### Sub-analysis of surveillance interval and clinical outcomes

Sub-analyses showed no significant differences in surveillance intervals based on risk factors, including the severity of LVLs or a history of head and neck cancer (Supplementary Table 2).

Furthermore, a sub-analysis comparing surveillance intervals is presented for the ≤ 6-month and > 6-month groups. Deep invasion was observed in 18% of cases in the > 6-month group and 7% of cases in the ≤ 6-month group. Non-curative resection rates were 21% and 12%, respectively. Additional treatment was required in 8% of cases in the > 6-month group and 0% of cases in the ≤ 6-month group. Detailed clinicopathological characteristics are presented in Supplementary Table 3.

### Cumulative incidence of secondary lesions in patients with multiple lesions

**Fig. 2:**
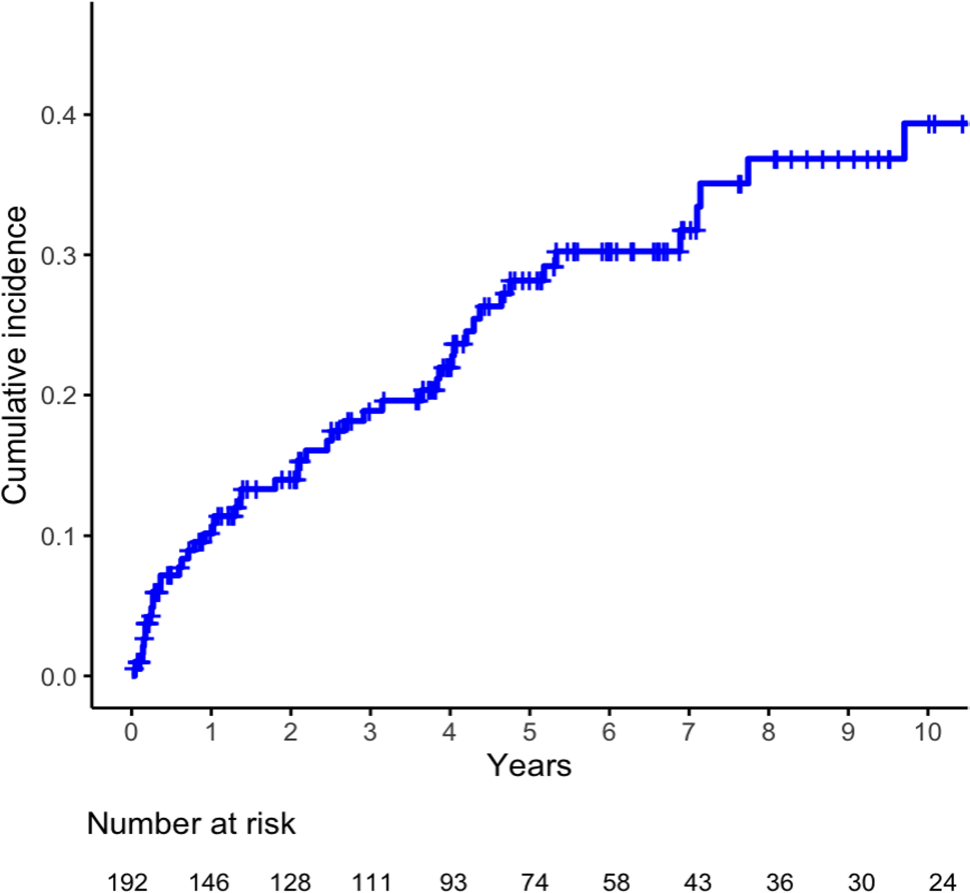
Cumulative incidence of secondary lesions in patients with multiple lesions. The cumulative incidence of secondary lesions was 10.2% (95% CI, 6.5–15.7), 18.9% (95% CI, 13.6–25.8), and 28.2% (95% CI, 21.5–36.4) at 1, 3, and 5 years, respectively. Number at risk is shown below the figure. *CI*, confidence interval

The cumulative incidence of secondary lesions in patients with multiple cancers is shown in Fig. 2. The incidence rates were 10.2% (95% CI: 6.5–15.7%), 18.9% (95% CI: 13.6–25.8%), and 28.2% (95% CI: 21.5–36.4%) at 1, 3, and 5 years, respectively.

## Discussion

In this study, we compared the clinicopathologic characteristics of initial and secondary cancers. Secondary ESCC exhibited a significantly lower proportion of tumors ≥ 20 mm (12% vs. 56%, *p* < 0.001) and a shallower depth of invasion (EP/LPM: 89% vs. 74%, *p* = 0.005) than initial lesions. To the best of our knowledge, no previous study has directly compared the characteristics of initial and secondary ESCC. These findings suggest that secondary lesions display distinct features.

Esophageal cancer frequently develops at different times due to field cancerization caused by smoking, alcohol consumption, and genetic or molecular biological factors [7]. Multiple heterogeneous occurrences have been reported, with a high cumulative incidence. Our results showed cumulative incidence rates of 10.2%, 18.9%, and 28.2% at 1, 3, and 5 years, respectively. These findings underscore the importance of careful, long-term surveillance in patients with a history of ESCC.

In this study, most secondary cancers were shallow lesions, classified as EP or LPM. Invasion depth is the most critical determinant in ESCC, as shallow invasion is generally associated with a low risk of metastatic recurrence. When submucosal invasion is identified, the lesion is classified as a non-curative resection, and additional treatments such as esophagectomy or chemoradiation therapy are recommended [20, 21]. Previous studies have shown that even in cases of pT1a-muscularis mucosae diagnosed from endoscopically resected specimens, when both lymphovascular invasion and vertical margin were negative, the 5-year metastatic recurrence rate was 5.6% [13]. In contrast, pEP/LPM lesions without vascular invasion have a minimal risk of lymph node metastasis and are defined as curative resections according to the current Japanese guidelines [14, 15]. Therefore, our results suggest that most secondary lesions, being shallow and negative for lymphovascular invasion, can be curatively managed with endoscopic resection alone, without the need for further invasive therapy. The finding that most secondary cancers were pEP/LPM suggests that, with appropriate surveillance, eradication therapy—such as that used in Barrett’s esophagus—may not be necessary.

Secondary carcinomas tended to be smaller than the initial lesions. When smaller lesions are detected at an early stage, a safer and more reliable curative resection can be achieved with ESD. Furthermore, in patients for whom ESD is difficult to perform, less invasive modalities such as endoscopic mucosal resection and APC may serve as effective treatment alternatives [22–25].

These favorable clinicopathological features—specifically, the smaller size and shallower depth of secondary lesions—can be attributed to several key factors. First, while initial lesions were identified through various means such as screening or symptom evaluation, all secondary lesions were detected during rigorous, scheduled post-ESD surveillance. Second, the routine use of high-definition modalities—including narrowband imaging [26], magnifying endoscopy, and Lugol’s iodine staining—greatly enhanced the detection of subtle, early-stage lesions. In addition, recent advances such as artificial intelligence-assisted diagnostic systems [27, 28] have made the detection of such minute lesions increasingly feasible in clinical practice. Finally, the heightened awareness of endoscopists regarding the risk of field cancerization ensured a more meticulous examination of the entire esophageal mucosa. This combination of proactive surveillance and diagnostic vigilance is crucial for improving the quality of life of patients and long-term prognosis.

Secondary cancers had a lower complication rate than initial cancers (12% vs. 34%, *p* < 0.001), particularly for esophageal stenosis (3% vs. 16%). This difference likely reflects the smaller size and circumference involvement of secondary lesions, which are closely associated with stenosis risk [29]. This finding suggests that endoscopic resection for secondary lesions can be performed with a lower risk of postprocedural complications, contributing to better long-term preservation of esophageal function.

ESCC arising in a background of multiple iodine-voiding lesions presents a significant challenge for post-treatment management. The high risk of multiple metachronous cancers after endoscopic resection requires careful follow-up [6]. According to the guidelines for ESD/endoscopic mucosal resection for esophageal cancer, the annual incidence of secondary cancers ranges from 2% to 9.2% [14, 15]. In our study, the median detection time for secondary lesions was 18 months, with a range extending up to 119 months post-treatment. These results emphasize the need for mid- to long-term surveillance. With a median follow-up interval of 5.25 months (range, 0.5–19 months), our analysis suggests that surveillance every 6 months can facilitate the detection of smaller, less invasive lesions. Although it did not reach statistical significance, our sub-analysis suggested a potential clinical trend where intervals exceeding 6 months were associated with a higher frequency of deep invasion and non-curative resection. Given the lack of sufficient data on longer intervals, such as 12 months, we cannot definitively conclude the optimal timing. However, this observed trend suggests that a 6-month interval provides a safer margin for detecting lesions at a curatively treatable stage. Future prospective studies are necessary to determine the optimal surveillance interval.

This study had several limitations. First, as a retrospective study conducted at a single institution, it may be prone to selection bias. In addition, some patients were lost to follow-up, potentially influencing the assessment of long-term outcomes. Second, follow-up intervals were not uniformly standardized, potentially affecting the timing of lesion detection. Therefore, a prospective observational study with standardized follow-up intervals (e.g., every 6 or 12 months) would be valuable for validating these findings. Third, pathological evaluation was not possible for lesions treated with APC, which may have limited the precise assessment of invasion depth in these cases. Fourth, this study did not include molecular or genomic analyses. Field cancerization in ESCC involves the clonal expansion of cells with *TP53* or *NOTCH1* mutations [30], and genetic predispositions like *ALDH2* and *ADH1B* polymorphisms significantly increase metachronous cancer risk [31]. While our clinical data highlight the benefits of rigorous surveillance, integrating molecular risk stratification remains a subject for future prospective investigations to further refine post-treatment management.

In conclusion, secondary lesions were detected at an earlier stage than the initial lesions, suggesting a high likelihood of achieving curative resection. Regular surveillance may enable effective management and early detection of secondary lesions.

## Supplementary Information

Below is the link to the electronic supplementary material.

Supplementary file1 (PDF 1735 KB)

Supplementary file2 (PDF 72 KB)

Supplementary file3 (PDF 85 KB)

Supplementary file4 (PDF 94 KB)

## Data Availability

The data that support the findings of this study are available on request from the corresponding author. The data are not publicly available due to privacy or ethical restrictions.
