# Supplementary material for: Clinical characteristics of synchronous and metachronous superficial esophageal squamous cell carcinoma during surveillance after endoscopic submucosal dissection
Source: Surg Endosc. 2026 Apr 20;40(6):4875–82. doi: 10.1007/s00464-026-12816-3 (PMC13246892; doi:10.1007/s00464-026-12816-3)
Supplement: Supplementary file 1 — Supplementary file1 (PDF 1735 KB) [file 464_2026_12816_MOESM1_ESM.pdf]

The STROCSS 2021 Guideline		
**Item no.**	**Item description**	**Page**
**TITLE**		
1	**Title**<br>• The word cohort or cross-sectional or case-control is included*<br>• Temporal design of study is stated (e.g. retrospective or prospective)<br>• The focus of the research study is mentioned (e.g. population, setting, disease, exposure/intervention, outcome etc.)<br><br>*STROCSS 2021 guidelines apply to cohort studies as well as other observational studies (e.g. cross-sectional, case-control etc.)	1
**ABSTRACT**		
2a	**Introduction** – briefly describe:<br>• Background<br>• Scientific rationale for this study<br>• Aims and objectives	4
2b	**Methods** - briefly describe:<br>• Type of study design (e.g. cohort, case-control, cross-sectional etc.)<br>• Other key elements of study design (e.g. retro-/prospective, single/multi-centred etc.)<br>• Patient populations and/or groups, including control group, if applicable<br>• Exposure/interventions (e.g. type, operators, recipients, timeframes etc.)<br>• Outcome measures – state primary and secondary outcome(s)	4
2c	**Results** - briefly describe:<br>• Summary data with qualitative descriptions and statistical relevance, where appropriate	4
2d	**Conclusion** - briefly describe:<br>• Key conclusions<br>• Implications for clinical practice<br>• Need for and direction of future research	5
**INTRODUCTION**		
3	**Introduction** – comprehensively describe:<br>• Relevant background and scientific rationale for study with reference to key literature<br>• Research question and hypotheses, where appropriate<br>• Aims and objectives	5
**METHODS**		
4a	**Registration**<br>• In accordance with the Declaration of Helsinki*, state the research registration number and where it was registered, with a hyperlink to the registry entry (this can be obtained from ResearchRegistry.com, ClinicalTrials.gov, ISRCTN etc.)<br>• All retrospective studies should be registered before submission; it should be stated that the research was retrospectively registered<br><br>** “Every research study involving human subjects must be registered in a publicly accessible database before recruitment of the first subject”*	N/A
4b	**Ethical approval**<br>• Reason(s) why ethical approval was needed<br>• Name of body giving ethical approval and approval number<br>• Where ethical approval wasn’t necessary, reason(s) are provided	10

4c	**Protocol**<br>• Give details of protocol (*a priori* or otherwise) including how to access it (e.g. web address, protocol registration number etc.)<br>• If published in a journal, cite and provide full reference	N/A
4d	**Patient and public involvement in research**<br>• Declare any patient and public involvement in research<br>• State the stages of the research process where patients and the public were involved (e.g. patient recruitment, defining research outcomes, dissemination of results etc.) and describe the extent to which they were involved.	N/A
5a	**Study design**<br>• State type of study design used (e.g. cohort, cross-sectional, case-control etc.)<br>• Describe other key elements of study design (e.g. retro-/prospective, single/multi-centred etc.)	6
5b	**Setting and timeframe of research –** comprehensively describe:<br>• Geographical location<br>• Nature of institution (e.g. primary/secondary/tertiary care setting, district general hospital/teaching hospital, public/private, low-resource setting etc.)<br>• Dates (e.g. recruitment, exposure, follow-up, data collection etc.)	6
5c	**Study groups**<br>• Total number of participants<br>• Number of groups<br>• Detail exposure/intervention allocated to each group<br>• Number of participants in each group	6
5d	**Subgroup analysis –** comprehensively describe:<br>• Planned subgroup analyses<br>• Methods used to examine subgroups and their interactions	N/A
6a	**Participants –** comprehensively describe:<br>• Inclusion and exclusion criteria with clear definitions<br>• Sources of recruitment (e.g. physician referral, study website, social media, posters etc.)<br>• Length, frequency and methods of follow-up (e.g. mail, telephone etc.)	6
6b	**Recruitment –** comprehensively describe:<br>• Methods of recruitment to each patient group (e.g. all at once, in batches, continuously till desired sample size is reached etc.)<br>• Any monetary incentivisation of patients for recruitment and retention should be declared; clarify the nature of any incentives provided<br>• Nature of informed consent (e.g. written, verbal etc.)<br>• Period of recruitment	6
6c	**Sample size –** comprehensively describe:<br>• Analysis to determine optimal sample size for study accounting for population/effect size<br>• Power calculations, where appropriate<br>• Margin of error calculation	N/A
**METHODS - INTERVENTION AND CONSIDERATIONS**		
7a	**Pre-intervention considerations –** comprehensively describe:<br>• Preoperative patient optimisation (e.g. weight loss, smoking cessation, glycaemic control etc.)<br>• Pre-intervention treatment (e.g. medication review, bowel preparation, correcting hypothermia/-volemia/-tension, mitigating bleeding risk, ICU care etc.)	N/A
