# Supplementary material for: Clinical characteristics of synchronous and metachronous superficial esophageal squamous cell carcinoma during surveillance after endoscopic submucosal dissection
Source: Surg Endosc. 2026 Apr 20;40(6):4875–82. doi: 10.1007/s00464-026-12816-3 (PMC13246892; doi:10.1007/s00464-026-12816-3)
Supplement: Supplementary file 2 — Supplementary file2 (PDF 72 KB) [file 464_2026_12816_MOESM2_ESM.pdf]

Supplementary Table 1. Details of clinical outcomes and management in patients with complications.

Complication	n	Management
Post-operative bleeding	15	Endoscopic hemostasis or conservative
Perforation	3	Endoscopic clipping or conservative
Pneumomediastinum	2	Endoscopic clipping or conservative
Esophageal stenosis	36	
- Managed endoscopically	35	Endoscopic dilation (balloon dilation and RIC)
- Managed surgically	1	Surgery (due to perforation during dilation)

Abbreviations: RIC, radial incision and cutting.
