# Supplementary material for: Clinical characteristics of synchronous and metachronous superficial esophageal squamous cell carcinoma during surveillance after endoscopic submucosal dissection
Source: Surg Endosc. 2026 Apr 20;40(6):4875–82. doi: 10.1007/s00464-026-12816-3 (PMC13246892; doi:10.1007/s00464-026-12816-3)
Supplement: Supplementary file 3 — Supplementary file3 (PDF 85 KB) [file 464_2026_12816_MOESM3_ESM.pdf]

Supplementary Table 2. Sub-analysis of surveillance intervals based on risk factors

	n (%)	Interval, median (IQR) (months)	*p*-value
Head and Neck Cancer, n (%)			0.173
History (–)	60 (61%)	6.25 (1.0–8.5)	
History (+)	39 (39%)	4.45 (2.1–6.7)	
LVL Grade, n (%)			0.321
Low-risk (A/B)	36 (37%)	4.80 (1.0–7.2)	
High-risk (C)	61 (63%)	5.50 (2.3–8.2)	

Abbreviations: IQR, interquartile range; LVL, Lugol-voiding lesion.

Note: Data on LVL grade were unavailable for two patients due to iodine allergy or omission of the Lugol's iodine test.
