# Supplementary material for: Clinical characteristics of synchronous and metachronous superficial esophageal squamous cell carcinoma during surveillance after endoscopic submucosal dissection
Source: Surg Endosc. 2026 Apr 20;40(6):4875–82. doi: 10.1007/s00464-026-12816-3 (PMC13246892; doi:10.1007/s00464-026-12816-3)
Supplement: Supplementary file 4 — Supplementary file4 (PDF 94 KB) [file 464_2026_12816_MOESM4_ESM.pdf]

Supplementary Table 3. Comparison of secondary lesions based on follow-up intervals.

	**≤ 6 months**	**> 6 months**	***p*-value**
Macroscopic Classification, n (%)			0.763
Protruding type	3 (6)	4 (10)	
Superficial type	18 (36)	15 (38)	
Excavated type	28 (56)	20 (50)	
Advanced type	1 (2)	1 (3)	
Location of Lesion, n (%)			0.955
Ce, Ut	6 (12)	6 (15)	
Mt	28 (56)	21 (53)	
Lt, Ae	16 (32)	13 (32)	
Diameter of ≥ 20 mm, n (%)	6 (12)	7 (18)	1.000
Circumference involvement ≥ 1/2 n (%)	13 (26)	8 (20)	0.618
Depth of invasion, n (%)			0.185
EP / LPM	40 (93)	33 (82)	
MM / SM	3 (7)	7 (18)	
HM, n (%)	2 (5)	3 (8)	0.668
VM, n (%)	0 (0)	1 (3)	0.481
LyV, n (%)	2 (5)	2 (5)	1.000
Non-curative resection, n (%)	5 (12)	8 (21)	0.370
Additional treatment, n (%)	0 (0)	3 (8)	0.084
Complications, n (%)	6 (12)	3 (8)	0.726

Abbreviations: Ce, cervical esophagus; Ut, upper thoracic esophagus; Mt, middle thoracic esophagus; Lt, lower thoracic esophagus; Ae, abdominal esophagus; EP, epithelium; LPM, lamina propria mucosa; MM, muscularis mucosae; SM, submucosa; HM, horizontal margin; VM, vertical margin; LyV, lymphovascular invasion.
